# Practical Notes and Formulæ

**Published:** 1884-01-20

**Authors:** 


### PRACTICAL NOTES AND FORMULÆ.

**Sweating in Phthisis.**—Speaking of the unsatisfactory results of all the remedies recommended for this condition, Dr. J. R. Forrest thus writes to the Lancet, October 27, 1883 :

I have found the following a most efficacious lotion :

℞. Sulphate of zinc........................ gr. iv,

Tincture of belladonna...................... ʒ j,

Water....................................... ℥ j.

The body to be sponged with the lotion at bed-time.

It has proved serviceable in my experience in cases both of the incipient and advanced disease, the excessive sweating being often quite restrained after two nights' sponging.—*Med. and Surg. Reporter.*

**External Application in Acne Rosacea.**—The following preparation is recommended by Helmayen :

℞. Slaked lime.............................. 1 part,
Sublimed sulphur........................... 2 parts,
Water...................................... 20 parts.

To be reduced by heat to twelve parts.

This is to be used for topical applications to the affected parts, and must, at first, be diluted with five parts of water, but gradually it may be used in more concentrated form.—*Ibid.*

**Croton Chloral in Whooping Cough.**—

℞. Croton chloral.......................... grs. xv,
Ether sulph................................ gtts. xx,
Bromid. potass............................. ʒ j,
Tr. belladonnæ............................. gtts. xv,
Tr. hyoscyami.............................. gtts. xxiv,
Syr. tolu q. s. to make.................... ℥ iv.

M. Sig. One teaspoonful every four hours until better, then only three times a day.

I have treated a number of cases, perhaps more than a hundred, since I first prescribed the medicine, and it has always acted very promptly in relieving the paroxysms, and I feel confident that those who will prescribe it will be pleased with the result.—Dr. E. A. Farquhar, in *Med. and Surg. Rep.*

**Lumbago.**—Prof. DaCosta cures lumbago with the following hypodermically :

Atropiæ sulph.............................. gr. 1-80,

Morphiæ sulph.............................. gr. ⅙. M.

—*College and Clinical Record.*

**Acute Tonsillitis.**—Dr. Maxwell, in Peoria Medical Monthly, says: The disease is usually ushered in by a chill, followed by fever. If seen in the first twenty-four hours I give the following prescription:

℞. Tr. guaiaci .......................... 4 drachms,
Spt. nit. dul .......................... 6 "
Tr. aconiti r., .......................... 8 drops,
Syr. aurant, q. s., .......................... 2 ounces.
M. Sig. A teaspoonful every two or three hours.

**Laville's Gout Mixture.**—An analysis of this medicine was published in the Berlin Industrie Blatter as follows:

| | | |
|---|---|---|
| Quinine | 0.5 | grammes, |
| Cinchonine | 0.6 | " |
| Colocynthine | 0.25 | " |
| Lime salts | 0.49 | " |
| Coloring matter | 0.3 | " |
| Alcohol | 10.0 | " |
| Water | 8.5 | " |
| Port wine | 80.0 | " |

**Injections for Gonorrhœa.**—

No. 1.—℞. Morph. sulph .......................... gr. jv.

Hydrat. chlor. .......................... ℈ ij,

Acid. boracici, sat. sol .......................... ℥ j.

Aq. camph., ad .......................... ℥ jv.

M. Sig. Inject.

No. 2.—℞. Tr. opii .......................... ℥ ss,
Acid carbol .......................... *m* v,
Ext. hydrast., fl. .......................... ℥ j,
Aq. rosarum, ad .......................... ℥ jv.
M. Sig. Inject.

No. 3.—℞. Hydrastin .......................... gr. v,
Potas. permang .......................... gr. viij,
Acidi tannici .......................... gr. xx,
Aq. rosarum, ad .......................... ℥ jv.
M. Sig. Inject.

No. 4.—℞. Ext. ergotæ, fl .......................... ℥ j,

Tr. opii .......................... ℥ ss,

Acid gallici .......................... gr. xx,

Aq. camph., ad. .......................... ℥ jv.

M. Sig. Inject.

Injection No. 1, seemed to give the greatest satisfaction, and was the longest used. At the expiration of six weeks, the patient getting more and more disgusted, and wishing to cease treatment, I tried the effect of corrosive sublimate, 1 gr. ℥ vj. At the end of three days from this treatment, he noticed a decided cessation of the discharge, and in eight days the discharge entirely ceased.—*Therapeutic Gaz.*

**Eczema of the Scalp in Infants.**—Dr. Lassar (Gaz. Med.) employs the following formula:

Salicylic acid.................................one part,
Tinc. of benzoin...............................two "
Vaseline.......................................fifty "

A certain quantity of this is smeared over the scalp two or three times a day, after having washed the infant's head with soap and water. To soften the crusts and facilitate the cleansing of the scalp, Dr. Lassar recommends the employment of oil containing two per cent. of salycilic acid.—*Med, Times.*

**Depilatories.**—The Turkish Rhusma (being employed by the voluptuous beauties in the harems, where etiquette demands *complete* nudeness of the body with the exception of the head, from which the hair is not removed) is composed of

Quicklime......................................50 parts,
Starch.........................................30 parts,
Orpiment....................................... 5 parts.

This is converted into a paste with water, and employed like the former preparation.—*Med. and Surg. Rep.*

**Dandruff.**—

℞ Aquæ.........................................℥ xij,

Listerine......................................℥ iv.

The above is the best preparation for dandruff and to promote growth of hair.—*Med. Brief.*

**Brandreth's Pills.**—Dear Sir: In your journal for November, p. 350, I notice two formulæ for Brandreth's pills, neither of which is correct. I inclose what I believe to be the true formula, and a most excellent pill it is:

℞ Ext. colocynth...............................℈ j,

Aloes socotrinæ................................℥ ij,

Gambogiæ.......................................℥ j,

Saponis castiliensis...........................℥ ss,

Ol. menthæ pip.................................gtt. ij,

Ol. cinnamomi..................................gtt. j,

Pulv. acaciæ, } ............................... aa q. s.

Alcoholis, }

Ut ft. pil. No. lxxx.

S. Dose, 1 to 3, as directed.

I often prescribe two of these pills with one three-grain mass hydrarg. pill at late bed-time, and obtain an admirable effect.

Very truly yours, D. S. CLARK, M.D.

—*American Druggist.*

**Paste or Glue for Paper Labels.**—(Said to make a first-class mucilage for gumming large sheets of paper, which may be kept for use without curling, and stick well on glass or other substances when wet):

Starch........................................2 drachms,
White sugar..................................1 ounce,
Gum arabic...................................2 drachms,
Water..........................................q. s.

Dissolve the gum, add the sugar, and boil until the starch is cooked.

Try also the following, said to be that used on postage stamps:

Gum dextrin..................................2 parts,
Acetic acid...................................1 part,
Water..........................................5 parts,
Dissolve in a water bath, and add alcohol........1 part.

—*Amer. Drug.*

**For Syphilis.**—

℞ Corrosive sublimate..........................gr. j,
Dilut. hydrochloric acid.....................gtts. xx,
Dissolve and add to comp. decoction sarsap'a..½ pt.

Dose, one teaspoonful three times a day.

**Tonic Bitters.**—

℞ Comp. tinc. gentian..........................ʒ viij,

Tinc. nux vomica..............................ʒ j,

Bi carb soda...................................ʒ ij.

M. Teaspoonful before each meal.

**Chronic Rheumatism and Neuralgia.**—

℞ Salicyllate of soda..........................2 drachms,
Simple elixir..................................2 ounces.

M. Dose, one teaspoonful four times in twenty-four hours.

**A Cure for Snake Bites.**--At the French Academy of Scicnces a paper was read concerning snake bites. It seems that one part of a solution of per manganate of potash, diluted with one hundred parts of water, if injected hypodermically near the bite, and also at every spot wherever swellings appear, is a sure antidote to the poison.

**Chilblains.**—

℞ Oil turpentine...............................℥ ij,

Spts. camphor..................................℥ ij,

Oil cajeput....................................ʒ j,

Tinc. opii.....................................℥ ij.

M. And apply as required.

